# A brief intervention is sufficient for many adolescents seeking help from low threshold adolescent psychiatric services

**DOI:** 10.1186/1472-6963-10-261

**Published:** 2010-09-06

**Authors:** Eila Laukkanen, Jukka J Hintikka, Jari Kylmä, Virve Kekkonen, Mauri Marttunen

**Affiliations:** 1 Department of Adolescent Psychiatry, Kuopio University Hospital and University of Kuopio, Kuopio, Finland; 2 Department of Psychiatry, Paijat-Hame Central Hospital and University of Tampere, Lahti, Finland; 3 Department of Health Sciences, University of Oulu, Senior Lecturer, Department of Nursing Science, University of Tampere, Finland; 4 Department of Adolescent Psychiatry, Joensuu Central Hospital, Finland; 5 Department of Adolescent Psychiatry Helsinki University Hospital and University of Helsinki, Finland; 6 Mental Health and Substance Abuse Services, National Institute for Health and Welfare, Helsinki, Finland

## Abstract

**Background:**

There has been a considerable increase in the need for psychiatric services for adolescents. Primary health care practitioners have a major role in detecting, screening and helping these adolescents. An intervention entitled SCREEN is described in this article. The SCREEN intervention was developed to help practitioners to detect and screen adolescent needs, to care for adolescents at the primary health care level and to facilitate the referral of adolescents to secondary care services in collaboration between primary and secondary health care. Secondly, the article presents the background and clinical characteristics of youths seeking help from the SCREEN services, and compares the background factors and clinical characteristics of those patients referred and not referred to secondary care services.

**Methods:**

The SCREEN intervention consisted of 1 to 5 sessions, including assessment by a semi-structured anamnesis interview, the structured Global Assessment Scale, and by a structured priority rating scale, as well as a brief intervention for each adolescent's chosen problem. Parents took part in the assessment in 39% of cases involving girls and 50% involving boys. During 34 months, 2071 adolescents (69% females) entered the intervention and 70% completed it. The mean age was 17.1 years for boys and 17.3 years for girls.

**Results:**

For 69% of adolescents, this was the first contact with psychiatric services. The most common reasons for seeking services were depressive symptoms (31%). Self-harming behaviour had occurred in 25% of girls and 16% of boys. The intervention was sufficient for 37% of those who completed it. Psychosocial functioning improved during the intervention. Factors associated with referral for further treatment were female gender, anxiety as the main complaint, previous psychiatric treatment, self-harming behaviour, a previous need for child welfare services, poor psychosocial functioning and a high score in the priority rating scale.

**Conclusions:**

A brief intervention carried out by a team including professionals from both primary and secondary level services was sufficient for a considerable proportion of adolescents seeking help for their psychiatric problems. Referral practices and counselling in special level services can be standardized. In the future, it will be important to develop and assess psychiatric services for adolescents using randomised controlled trials.

## Background

Adolescent development with all the developmental "landmarks", such as separation from the parents, acquiring an adult personal sexual identity and the search for adult goals in life, as well as physical changes related to hormonal and sexual maturation, increase the vulnerability of adolescents to psychiatric symptoms, most commonly to depressive symptoms. Epidemiological studies have shown that the incidence and prevalence of psychiatric disorders increase during adolescence, and many adult psychiatric disorders have their onset during this period [1-4]. Thus, the early detection and intervention of psychiatric symptoms and disorders may have a major impact in preventing psychiatric disorders in adulthood. Despite the relatively high prevalence of psychiatric disorders (estimated at 15 to 25%), psychopathology in adolescents tends to be unrecognized and under-treated [5,6]. Compared to psychiatric disorders among children, disorders among adolescents are commonly undetected by parents and teachers [7].

Health care centres in primary care have a major role in detecting and screening patients with psychiatric problems in Finland. General practitioners are in a good position to provide services, but their knowledge and skills to assess and intervene in adolescent psychiatric problems are often inadequate [8-11]. The roles of primary care and secondary care services in treating adolescent psychiatric problems are often unclear [8,9,12,13], leading to delays in referring youths for psychiatric evaluation. Furthermore, the threshold for a referral to psychiatric services seems high for adolescents [14].

**Figure 1:**
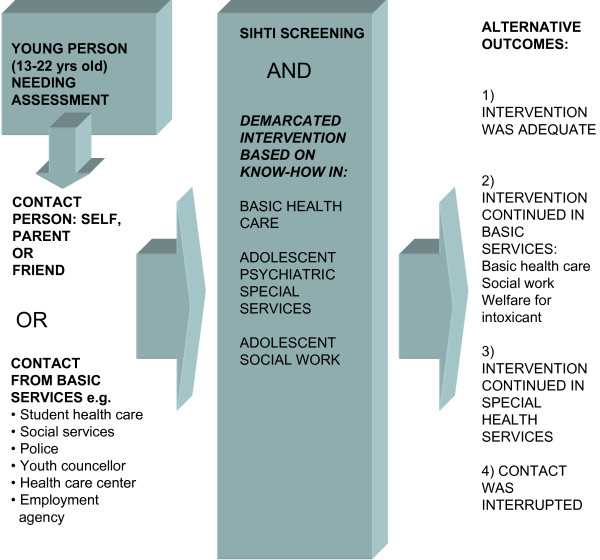
**The concept of SCREEN intervention**.

Finnish people have universal access to health care, including adolescent psychiatric care. There has been a considerable increase in the need for psychiatric services for adolescents in Finland [14]. In order to develop and improve the health care system for adolescents who have psychosocial problems, two Finnish Health Districts in three regions (Kuopio with a population of 90 000, Lappeenranta with a population of 59 000 and Imatra with a population of 30 000) started a development project entitled SCREEN intervention (SIHTI in Finnish). SCREEN was based on collaboration between primary health care and secondary care adolescent psychiatric services. The leading principles were that the services should be easy to access without a referral, and that the psychosocial situation of the adolescents and their need for further psychiatric care would be evaluated during a brief intervention consisting of 1 to 5 sessions. The concept of SCREEN intervention is described in Figure 1.

The aims of the project were to develop problem identification and treatment practices in primary health care so that minor adolescent problems could be treated in primary care, and on the other hand to facilitate referral to secondary care services when needed in collaboration between primary and secondary health care.

The aims of this article are to describe the SCREEN intervention, present the background and clinical characteristics of youths seeking help from the SCREEN services, and to compare the background factors and clinical characteristics of those patients referred and not referred to secondary care services.

## Methods

### Intervention

In each region, a team including professionals from both primary care (GP, school nurse, social worker) and secondary care adolescent psychiatric services (psychologist, psychiatric nurse, consulting adolescent psychiatrist) was formed. When constituting these teams, working experience with adolescents/families and therapeutic training were prioritised. The SCREEN intervention consisted of an evaluation of the adolescents' living circumstances and assessment of the severity of problems during 1 to 5 sessions. The population and co-workers in schools and the health care system were informed about these services beforehand via announcements. Adolescents or their parents were advised to telephone or come directly to the SCREEN office.

**Figure 2:**
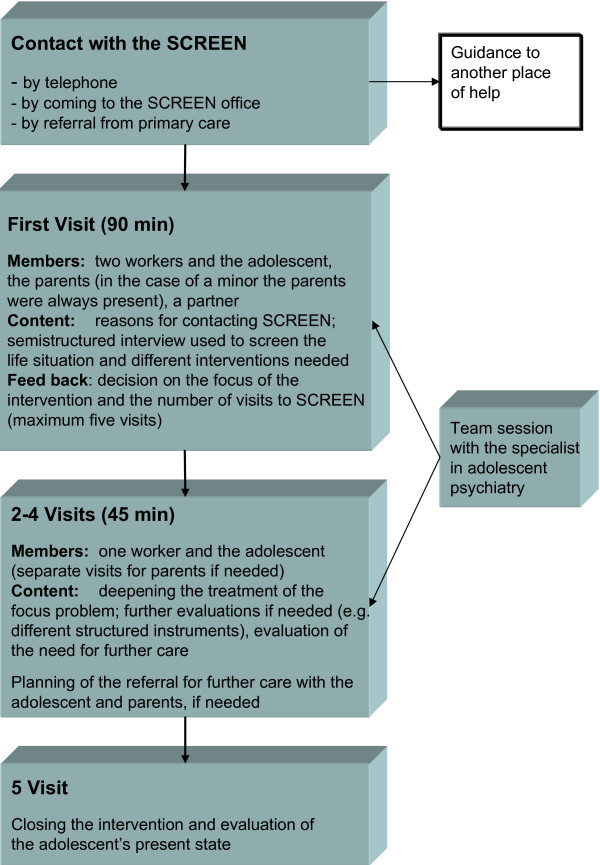
**Description of the SCREEN intervention**.

**Figure 3:**
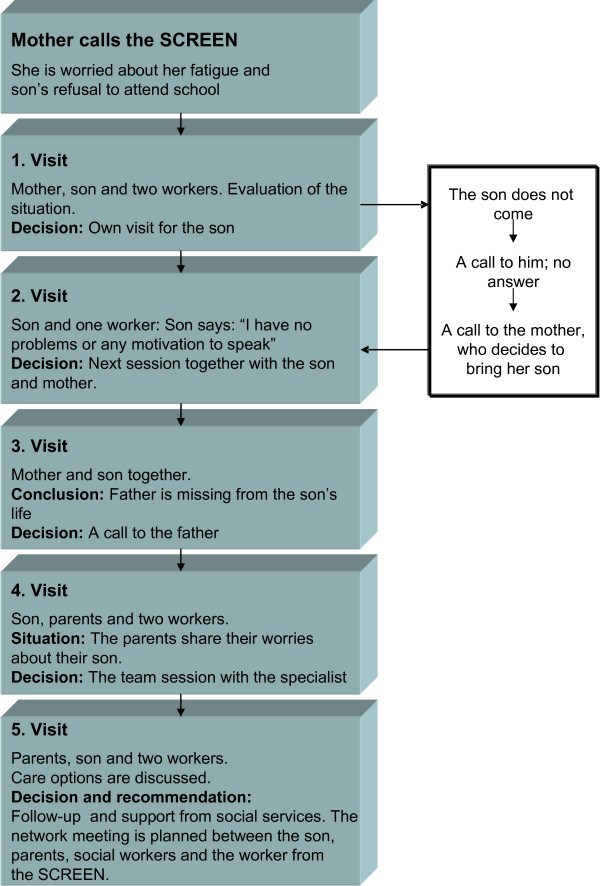
**A case example**.

The content of SCREEN intervention is described in Figure 2. During the first telephone contact the adolescent or parent was interviewed using a brief, semi-structured interview schedule. Based on this interview, a plan on who would participate in the first face-to-face interview (the adolescent alone or with parents) was made. Parents were asked whether they were willing to take part in the intervention. The first evaluation session (lasting 90 minutes) was conducted by two team members, and during this session the focus and time limits for intervention were decided in collaboration with the adolescent and parents. The following sessions (each 45 minutes) were carried out by one team member alone with the adolescent. Treatment schedules were individualized from a range of psychosocial interventions, including assessment and supportive intervention, brief individual psychotherapy, and psychotropic medication (e.g. antidepressive medication prescribed by a GP after consulting the psychiatrist) when appropriate. School personnel and/or child welfare personnel were also asked to participate in the intervention when appropriate. The final assessment of the adolescent's psychosocial situation and psychiatric problems as well as referral to secondary care services was made in a team session where all team members (nurses, GP, psychologist and social worker) took part and where the consulting adolescent psychiatrist also participated. A case example is presented in Figure 3.

### Subjects

In total, 2071 youths consecutively entered the SCREEN services during the study period from 1 May 2005 to 30 December 2008 (34 months). Of these, 56.8% were evaluated in Kuopio, 31.1% in Lappeenranta and 12.1% in Imatra. The intervention was completed by 1456 subjects (70.3%). The study was conducted in accordance with the Declaration of Helsinki and was approved by the Ethical Committee of Kuopio University Hospital and University of Kuopio (144//2004). The data presented here were collected from health registers formed for SCREEN intervention in Kuopio and in Lappeenranta. The head doctors granted permission to use the local registers.

### Study Procedure

The medical staff of the project were trained to perform all assessments in a standardized manner for evaluation and research purposes. All subjects were interviewed using a semi-structured checklist. This checklist was formed for clinical purposes in order to standardize the information collected from adolescents and parents. During the interview, data were collected on the adolescents' socio-demographic and socio-economic background and academic achievements, the main reason for seeking help, the person who first contacted the service, previous contacts with health or social services due to current problems, previous psychiatric treatment and previous or current contact with social services. Data on the adolescents' self-harming behaviour were also collected. Each subject was asked whether he or she had ever had suicidal thoughts (no/yes, at the moment/yes, previously) and had ever attempted suicide (yes/no). (See Table 1).

**Table 1:** Sociodemographic characteristics of adolescents seeking help from low-threshold walk-in clinics

	Study subjects		
	**Girls** **n = 1429**	**Boys** **n = 642**	**P value**

Age, mean (SD)	17.3 (2.6)	17.1(2.6)	0.04^1^
Range	10.9-23.7	9.6-23.1	
First contact from (%)			< 0.001^2^
Adolescent her-/himself	36.2	19.5	
Parent	25.2	31.4	
School (health care) personnel	22.3	20.8	
Psychiatric care personnel	3.6	3.1	
Other	12.5	13.7	
Education (%)			< 0.001^2^
Comprehensive School	47.2	56.1	
High School	19.7	14.5	
Vocational School/Secondary education	23.2	18.1	
College/University	8.2	4.5	
Dropped out of school	1.7	6.9	
Occupation (%)			< 0.001^2^
Student	87.6	78.7	
Employed	5.7	6.7	
Unemployed	2.6	5.1	
Other	4.1	9.5	
Form of dwelling (%)			< 0.001^2^
With parents	57.0	71.3	
Own household	33.9	18.2	
Other	9.1	10.4	

^1 ^Mann-Whitney U-test, ^2 ^Chi-squared test.

The psychosocial functioning of the subjects at entry and at the end of the intervention was assessed using the structured Global Assessment Scale (GAS) [15]. This scale is used throughout Finland in specialized adolescent psychiatric services. The GAS is a 100-point single-item observer-rating scale rating psychosocial functioning on a hypothetical continuum from excellent to extremely poor. Scores on the scale, which range from 0 (poor) to 99 (excellent), are divided into 10 ranges of functioning. A written description of each 10-point interval covers both symptom severity and social and occupational functioning. The GAS provides a summary score indicating the level of the subject's overall psychosocial functioning. All adolescents entering the SCREEN were also assessed by the team members using the structured priority criteria tool for elective secondary care adolescent psychiatric services [16]. This rating tool has been modified for use in Finland from the West Canada Waiting List Project [17]. The maximum total score of the tool is 100 points; a score of 50 is the cut-off point for specialized psychiatric services for adolescents. The priority rating tool comprises 15 items organized in four blocks: (1) symptoms and risks, (2) impaired functioning, (3) additional risk factors and (4) expected prognosis without treatment [16].

### Data Analysis

The chi-squared test and Mann-Whitney U-test were used to describe the subjects and to compare male and female adolescents as well as those who did or did not complete the intervention (loss analysis). These tests were also used to analyze differences between adolescents who did or did not need further support and treatment. Normality and homoscedasticity were visually checked.

Finally, multivariate regression models were constructed to determine which variables associated with the need for further treatment. Variables showing statistical significance (p < 0.05) in univariate analyses as well as the study centre were included in the multivariate stepwise logistic regression models. The results were expressed as ORs (odds ratios) with their 95% confidence intervals. All statistical analyses were performed with the statistical package SPSS for Windows 14.0.

## Results

### Sociodemographic and clinical characteristics at entry

Altogether, 1429 (69%) females and 642 (31%) males entered the SCREEN services. All adolescents were white Caucasians. Female participants were statistically significantly older than male participants. The first person contacting the services was most commonly the adolescent herself among females and a parent among males. Most adolescents were students, females more commonly than males. Approximately half of the adolescents were studying in comprehensive school. Females were more commonly studying in high school or at university. Unemployment was more common among males than females. Boys more often lived with their parents than girls and, vice versa, girls more commonly lived in their own household (Table 1). Nearly half of the study subjects had divorced parents, boys more often than girls (46.7% vs. 42.8%, p = 0.01).

The most common reasons for seeking help from the SCREEN services were depressive and anxiety symptoms, especially in girls. Among males, problems at school or work, and antisocial or violent behaviour also were common reasons for help seeking. Among females, problems in social relationships also were common (Table 2). Eating problems as a main complaint were more common in girls than in boys (3.5% vs. 0.3%, p < 0.001). Over a third of all subjects had previously received psychiatric treatment, and a sixth had had contacts with child welfare services. Nearly 70% of the subjects had contacted certain services due to their current problems, most commonly school services or public health care services. Previous or current self-harming behaviour were more common among females than males. Nearly 4% of the subjects had attempted suicide during their lifetime, with no gender difference (Table 2).

**Table 2:** Clinical characteristics of adolescents seeking help from low-threshold walk-in clinics

	Study Subjects		
	**Girls** **n = 1429**	**Boys** **n = 642**	**P value**

Previous psychiatric treatment (%)	30.9	36.6	0.65^1^
Previous contact with child welfare services (%)	13.8	25.2	0.02^1^
Previous contacts due to current problems (%)			< 0.001^1^
No previous contacts	38.9	30.2	
Psychiatric care	5.0	4.4	
Public health care	26.2	18.2	
Child welfare services	6.0	6.9	
School	26.2	33.3	
Other	5.2	6.9	
Self-harming behaviour (%)	24.7	16.4	< 0.001^1^
Suicide attempt (%)	3.9	4.7	0.58^1^
The main reason for contact (%)			< 0.001^1^
Sleeping problems	4.1	3.1	
School/Work problems	9.5	21.5	
Depressive symptoms	34.2	24.1	
Anxiety symptoms	15.3	15.0	
Problems in social relationships	16.1	9.3	
Self-harming behaviour	2.0	0.8	
Antisocial/violent behaviour	3.8	11.1	
Eating problem	3.4	0.3	
Substance abuse/dependence	2.1	4.8	
Traumatic experiences	3.7	1.9	
Other psychiatric symptoms^2^	5.8	8.1	

^1 ^Chi-squared test. ^2 ^Including one case with psychotic symptoms.

The mean number of therapy sessions was 3.8 among girls and 3.5 among boys. Parents took part in the assessment more commonly among males than females. During the current assessment period, 11% of the subjects had been in contact with child welfare services. Psychosocial functioning in girls measured by the GAS score was better than that of boys, both at the time of entry and at the end of the SCREEN intervention. Accordingly, boys had higher priority rating scale scores than girls (Table 3). Girls successfully completed the intervention more commonly than boys (73.1% vs. 64.2%, p < 0.001).

**Table 3:** Psychosocial evaluation during SCREEN intervention.

	Study Subjects		
	**Girls** **n = 1429**	**Boys** **n = 642**	**P value**

Number of sessions (Mean (SD))	3.8 (2.3)	3.5 (2.2)	0.001 ^1^
Number of individual sessions (Mean (SD))	2.9 (1.9)	2.5 (1.8)	< 0.001^1^
Parents took part in the assessment (%)	39.0	50.0	< 0.001^1^
Contact with child welfare services during assessment (%)	10.4	13.2	0.06 ^2^
Priority rating scale score (Mean (SD, CI))	38.4 (24.0; 37.1-39.6)	42.6 (25.0; 40.7-44.6)	0.001 ^1^
GAS at entry (Mean (SD, CI))	56.5 (9.4; 56.0-56.9)	54.5 (11.2; 53.7-55.4)	< 0.001^1^
GAS at end (Mean (SD, CI))	60.3 (11.4; 59.7-60.9)	58.3 (12.5; 57.4-59.3)	< 0.001^1^

^1 ^Mann-Whitney U-test. ^2 ^Chi-squared test.

### Characteristics of those who did or did not complete the intervention

Girls completed the intervention more often than boys (73.1% vs. 64.2%, p < 0.001). There was a significant difference between centres in the proportion of those who completed the intervention: 68.1% in Kuopio, 71.4% in Lappeenranta and 78.0% in Imatra (p = 0.006). Those whose parents participated in the intervention were more often completers than others (84.9% vs. 59.6%, p < 0.001). Completing the intervention was more common among those who had sleeping problems (74.4%), depressive symptoms (73.8%), anxiety symptoms (78.4%) or self-harming behaviour (78.8%) as the main reason for contact. Conversely, completing the intervention was less common among those with school or work problems (61.3%), substance abuse or dependence (67.2%) or traumatic experiences (64.6%) as the main reason for contact (p < 0.001 for overall differences in proportions). Finally, completers had higher GAS scores at entry than non-completers (mean 56.6 (SD 9.6) versus 54.2 (SD 10.7), p < 0.001). No difference was found in the priority rating scale total score between the groups (mean 39.6 (SD 26.9) versus 40.1 (SD 17.1), respectively; p = ns.).

### Characteristics of adolescents referred for further treatment

Of the 1456 study subjects who completed the SCREEN intervention, 913 (62.7%) were referred for further treatment by secondary care services (including psychiatric services, services for substance abuse and follow-up sessions in child welfare services). Thus, the SCREEN intervention was sufficient for 37.3% of those who completed it. The referral decision was reached in collaboration with the adolescent, parents (if participating) and the team. The proportion of those who needed referral differed between centres: 65.3% in Kuopio, 52.8% in Lappeenranta and 75.4% in Imatra (p < 0.001). When the subjects referred for further treatment and those not referred were compared, no statistically significant gender differences were found between the two groups, but those who required further treatment were older. A higher educational level, parents not as the persons initiating contact with the SCREEN service, and depression and anxiety as the main reason for contact were more common among those who were referred for further treatment. Moreover, previous psychiatric treatment, contacts with child welfare services, self-harming behaviour and suicide attempts were more common among those referred for further treatment. Psychosocial functioning, both at entry and at the end of the SCREEN intervention, was statistically significantly worse and the priority rating scale scores higher among those referred for further treatment (Table 4).

**Table 4:** Demographic and clinical characteristics of adolescents according to referral for further treatment among those who completed the intervention

	Need for further treatment		
	**No** **n = 543**	**Yes** **n = 913**	**P value**

Age, mean (SD)	16.9 (2.7)	17.4 (2.7)	< 0.001 ^1^
Sex (%)			
Girls	70.9	72.2	0.60 ^1^
Education (%)			0.001 ^1^
Comprehensive school	54.5	47.1	
High School	21.7	19.4	
Vocational School/Secondary education	15.3	24.4	
College/University	7.7	8.3	
Dropped out of school/Not known	0.7	0.8	
Occupation (%)			0.08 ^2^
Student	90.1	85.4	
Employed	5.2	7.0	
Unemployed	2.0	3.1	
Other	2.8	4.5	
Referral (%)			0.02 ^2^
Adolescent	33.7	32.1	
Parents	36.5	30.7	
School personnel	17.7	19.5	
Psychiatric care personnel	1.8	3.6	
Other	10.3	14.1	
Previous contacts caused by current problems (%)			< 0.001 ^2^
No previous contacts	45.7	34.7	
Psychiatric care	3.3	5.9	
Public health care	20.1	26.9	
Social worker	5.5	6.1	
School	20.8	20.5	
Other	4.6	5.8	
The main reason for contact (%)			< 0.001 ^2^
Sleeping problems	5.0	3.4	
School/Work	12.5	11.0	
Mood	27.8	35.5	
Anxiety	14.2	18.6	
Relationships	18.6	11.3	
Self-harming behaviour	0.9	2.3	
Antisocial/violent behaviour	6.8	5.8	
Eating problem	1.8	2.8	
Addiction	2.9	2.7	
Traumatic experiences	4.6	1.9	
Other psychic symptoms ^3^	4.8	4.7	
Self-harming behaviour (%)	13.3	32.0	< 0.001 ^2^
Suicide attempt (%)	0.9	5.4	< 0.001 ^2^
Previous psychiatric treatment (%)	21.4	37.3	< 0.001 ^2^
Parents took part in assessment period (%)	49.2	52.5	0.22 ^2^
Previous contact with child welfare services (%)	10.1	17.7	< 0.001 ^2^
Contact with child welfare services in this assessment (%)	5.7	16.8	< 0.001 ^2^
GAS at entry (Mean (SD))	61.7 (8.3)	53.5 (9.0)	< 0.001 ^1^
GAS at the end (Mean (SD))	69.3 (8.4)	56.1 (10.4)	< 0.001 ^1^
Priority rating scale score (Mean (SD))	24.3 (21.9)	48.6 (25.5)	< 0.001 ^1^

^1 ^Mann-Whitney U-test. ^2 ^Chi-squared test. ^3 ^Including one case with psychotic symptoms.

### Factors associated with referral for further treatment

In the final logistic regression model, the statistically significant predictors of referral for further treatment among those who had completed the intervention were female gender, anxiety as the main complaint, previous psychiatric treatment, self-harming behaviour, a previous need for child welfare services, poor psychosocial functioning at entry and a high score in the priority rating scale (Table 5). In this group, the priority rating scale score was over 50 in 675 (46.4%) subjects and 80.7% of them were referred for further treatment (p < 0.001). The GAS score at entry was less than 50 in 243 (24.0%) subjects and 90.1% of them were referred for further treatment (p < 0.001).

**Table 5:** Factors associated with referral for further treatment ^1^

Variable	aOR (95% CI)	P value
Girls vs. boys	1.41 (1.06-1.87)	0.019
Anxiety as the main complaint (yes/no)	1.59 (1.13-2.23)	0.008
Self-harming behaviour (yes/no)	1.75 (1.26-2.43)	0.001
Previous psychiatric treatment (yes/no)	1.69 (1.24-2.18)	0.001
Previous contact with children welfare services (yes/no)	1.80 (1.21-2.67)	0.004
GAS total score at entry	0.95 (0.93-0.97)	< 0.001
Priority rating scale total score	1.03 (1.02-1.04)	< 0.001

^1 ^Adjusted for the centre.

aOR = adjusted odds ratio. CI = Confidence interval.

## Discussion

The SCREEN service reached 2071 adolescents, more females than males, during the three-year study period. Most of them were students at comprehensive school living with one or two biological parents. The most common reasons for contacting the services were symptoms of depression and anxiety. For about two thirds of the help-seeking adolescents, the SCREEN was their first contact with psychiatric services. The brief intervention was sufficient for approximately 40% of the contacting adolescents. Female gender, previous psychiatric or child welfare contacts, suicidal tendencies and poor psychosocial functioning characterized those subjects who were referred for specialized services.

The finding that the proportion of females entering the SCREEN service was twice that of males and that females contacted the service on their own initiative more commonly than males accords with previous research suggesting that seeking help for psychiatric problems may be easier for adolescent females than males [18]. On the other hand, male subjects more commonly lived with their parents and their educational level was lower than that of females. It is possible that the recognition of mental health problems is poorer in these families. Zachrisson et al. [19] also concluded in their study that the poor recognition of mental health problems in adolescents or their unwillingness to seek help for these problems are the major hindrances restricting treatment.

In accordance with epidemiological research on adolescent psychiatric problems [20], females in this study sought help more often for internalizing problems such as depressive or anxiety symptoms or problems in social relationships, while boys entered the SCREEN more often due to externalizing problems such as problems at school or work or antisocial behaviour. Previous studies have also suggested that girls suffering from depression are more active in seeking help than boys [21].

Although the SCREEN intervention was kept very brief, consisting of only three to five appointments, the psychosocial functioning of the subjects improved. This finding suggests that even very brief interventions combined with spontaneous remission are sufficient for many adolescent psychiatric problems, as previously reported by Andrade et al. [22].

Not surprisingly, previous psychiatric treatment, self-harming behaviour, a need for child welfare services and poor psychosocial functioning were associated with referral for further treatment. These results indicate that a brief intervention is not sufficient for adolescents with multiple, long-lasting and serious problems. It also appears that the priority rating scale for elective secondary care adolescent psychiatric services successfully screens these adolescents, as previously reported by Kaltiala-Heino et al. [16].

Female gender was a predictor for needing further psychiatric services. Many factors could explain this result. Boys had more externalising symptoms than girls and they more often came on the initiative of their parents. One explanation may also be that the SCREEN intervention was not successful in motivating boys with externalizing symptoms to use adolescent psychiatric services. It seems easier to offer referral for psychiatric services for a girl who has internalising symptoms and is motivated to seek treatment.

The study sample was a large, unselected sample from two Finnish health districts, representing adolescents from urban and semi-urban areas. Data were collected via a semi-structured clinical interview and reliable structured ratings. The main limitation was the lack of use of a structured diagnostic interview, precluding analysis of formal psychiatric diagnoses of subjects in need of further treatment. Generalization of the results to other cultures should take into account possible differences in health care systems. Future analyses of these data need to include a follow-up of youths referred and not referred for further treatment after the brief intervention.

## Conclusions

A brief intervention, tailored individually according to the needs of each adolescent, was sufficient at this stage of life for a considerable proportion of those contacting the SCREEN service. The decision on referral to further treatment in cooperation with the adolescent/parent could be appropriately made during the intervention. Bringing together knowledge from specialist level adolescent psychiatric services and primary care services seems to have been successful. It appears possible and appropriate to assess and in many cases to treat adolescent psychiatric problems in primary health care without referral to a specialist. The SCREEN service most probably lowers the threshold for seeking help and helps to avoid youths being labelled for using mental health services [21]. The intervention also shows that referral practices and counselling in specialist level services can be standardized. However, it must be noted that only after a randomised, controlled study can final conclusions be made about the effect of intervention. In the future it will be important to develop psychiatric services for adolescents at all levels of the health care system in order to intervene early in psychiatric disorders. In particular, boys with externalizing problems are a great challenge for the health care system.

## Competing interests

The authors declare that they have no competing interests.

## Authors' contributions

EL planned the intervention, participated in the planning of the study and in the preparation and writing of the manuscript; JH performed the statistical analysis of the study and participated in writing the manuscript; JK participated in the planning of the project and in the preparation and writing of the manuscript; VK participated in the preparation and writing of the manuscript; MM participated in the writing of the manuscript.

## Funding

This SCREEN intervention and study received funding from the Ministry of Social Affairs and Health of Finland and Kuopio University Hospital.

## Pre-publication history

The pre-publication history for this paper can be accessed here:

http://www.biomedcentral.com/1472-6963/10/261/prepub
